# White matter hyperintensities, retinal vascular calibre and changes in age-related hearing loss

**DOI:** 10.1093/braincomms/fcag133

**Published:** 2026-04-15

**Authors:** David P Q Clark, Cammie Tran, Sultana Monira Hussain, Catherine Robb, Carlene Britt, Robyn L Woods, Paul A Yates, Amy Brodtmann, Mohamed Salah Khlif, Geoffrey Donnan, Gary Rance, John J McNeil

**Affiliations:** School of Public Health and Preventive Medicine, Monash University, Clayton, Melbourne, Victoria 3004, Australia; School of Public Health and Preventive Medicine, Monash University, Clayton, Melbourne, Victoria 3004, Australia; School of Public Health and Preventive Medicine, Monash University, Clayton, Melbourne, Victoria 3004, Australia; Department of Medical Education, The University of Melbourne, Melbourne, Victoria 3010, Australia; School of Public Health and Preventive Medicine, Monash University, Clayton, Melbourne, Victoria 3004, Australia; School of Public Health and Preventive Medicine, Monash University, Clayton, Melbourne, Victoria 3004, Australia; School of Public Health and Preventive Medicine, Monash University, Clayton, Melbourne, Victoria 3004, Australia; Department of Geriatric Medicine/Aged Care Services, Continuing Care Department, Austin Health, Heidelberg, Victoria 3084, Australia; Department of Medicine, Austin Health, the University of Melbourne, Heidelberg, Victoria 3084, Australia; Department of Neuroscience Central Clinical School, Monash University The Alfred Centre,Melbourne, Victoria 3004, Australia; Department of Neuroscience Central Clinical School, Monash University The Alfred Centre,Melbourne, Victoria 3004, Australia; Melbourne Brain Centre, University of Melbourne, Department of Neurology, Royal Melbourne Hospital, Melbourne, Victoria 3010, Australia; Department of Audiology and Speech Pathology, The University of Melbourne, Melbourne, Victoria 3010, Australia; School of Public Health and Preventive Medicine, Monash University, Clayton, Melbourne, Victoria 3004, Australia

**Keywords:** ageing, cerebral small vessel disease, retinal vascular calibre, age-related hearing loss, white matter hyperintensities

## Abstract

Cerebral small vessel disease may contribute to age-related hearing loss pathogenesis by reducing blood flow to the cochlea and/or areas of the brain important for hearing. Cerebral white matter hyperintensities (WMHs) and retinal vascular calibre are both considered to be non-invasive markers of cerebral small vessel health. This study investigated the relationship between age-related hearing loss, retinal vascular calibre and WMHs in older adults using data from the ASPirin in Reducing Events in the Elderly (ASPREE) trial, including participants who underwent hearing assessment, retinal photography and brain magnetic resonance imaging. Participants were free of evident cardiovascular disease at recruitment between 2010 and 2014. Retinal vascular calibre was measured using central retinal arteriolar and venular equivalents. Total brain WMH volumes were calculated using an automated lesion prediction algorithm and further segmented as deep WMH and periventricular WMH. Hearing acuity was assessed using pure tone audiometry and speech perception in background noise. A total of 308 participants (aged 70+) were included. In both cross-sectional and longitudinal analyses no associations were found between baseline central retinal arteriolar equivalent, central retinal venular equivalent, total WMH, deep WMH or periventricular WMH in relation to changes in audiometric 0.5, 4 and 8 kHz thresholds or speech perception after adjusting for confounding factors. This study identified no relationship between retinal vascular calibre, WMH volumes and hearing function in healthy older adults. This suggests that microvascular changes in the eye and brain may occur independently of changes in auditory function in older individuals.

## Teaser

Clark *et al*. report that retinal vascular calibre and white matter hyperintensity volume are not associated with hearing acuity or its 3-year change in older adults. This suggests microvascular changes in the eye and brain may occur independently of auditory function in ageing.

## Graphical Abstract

**Graphical Abstract:**
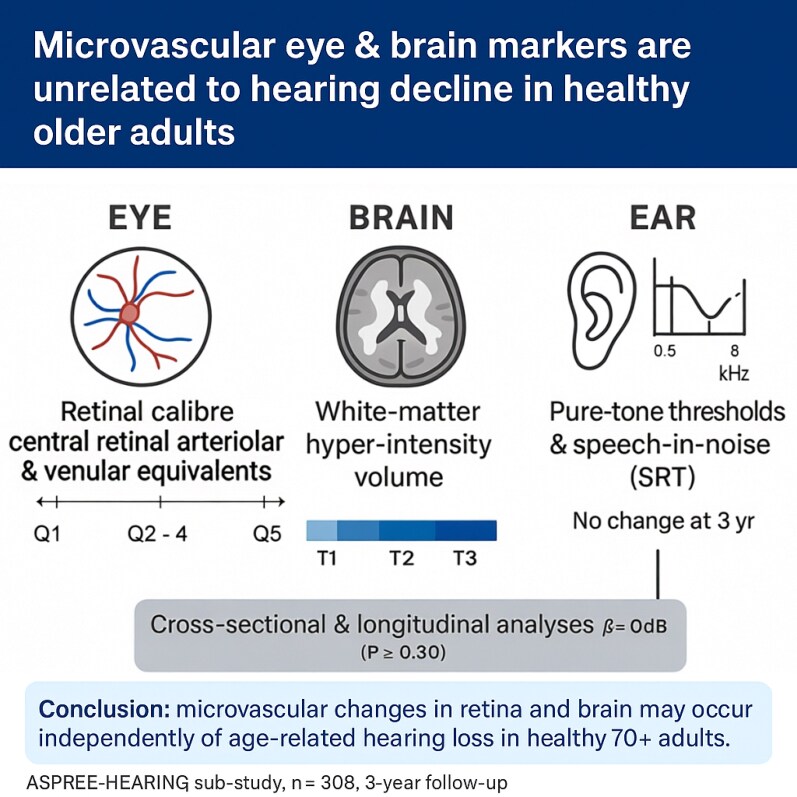

## Introduction

Age-related hearing loss (ARHL) affects approximately 50% of adults aged 70 years or above and impacts both socialization and quality of life.^1-3^ The early stage of ARHL involves degenerative changes in the cochlea with a later effects on neuronal pathways important for hearing.^4^ Evidence from animal studies suggests that microvascular pathology may contribute to the pathogenesis within the cochlea by reducing blood flow to the stria vascularis, an area essential for normal cochlear function.^5^ Known risk factors for ARHL include cigarette smoking and type 2 diabetes, both of which involve pathological changes in small blood vessels.^6-8^

Cerebral small vessel disease is believed to underpin the presence of white matter hyperintensities (WMH), a common finding on MRI scans of the brain in older individuals.^9^ The pathological basis of WMHs includes demyelination and disruption of axonal tracts and is more common amongst those with vascular ‘risk factors’ including hypertension, diabetes and cigarette smoking.^10-14^ The small blood vessels of the eye are considered to be an extension of the cerebral vasculature and provide an opportunity to visualize microvasculature in a non-invasive manner. If either retinal vascular or cerebral white matter characteristics showed a strong association with ARHL it might provide an opportunity for early diagnosis or a better understanding of its causes.

There is currently limited information on the relationship between retinal vascular indices, the extent of cerebral WMHs and the severity of ARHL.^15-18^ Published studies concentrated on younger persons, been contradictory or had methodological limitations. The ASPirin in Reducing Events in the Elderly (ASPREE) trial provides an opportunity to investigate the association of both retinal and WMHs with the severity of ARHL in a population of older men and women. The study benefits from preplanned, objective measurements in a large cohort of relatively healthy individuals.

## Materials and methods

### Study design and setting

Australian and US adults aged 70 years and over (aged 65 years for US minorities) participated in the ASPREE clinical trial which investigated the effects of a daily 100 mg enteric coated aspirin dose versus placebo on disability-free survival.^19^ Exclusion criteria included established cardiovascular disease, independence-limiting physical disability or cognitive impairment or conditions likely to cause death within 5 years. Australian ASPREE participants were also invited to participate in sub-studies involving MRI neuroimaging (the ASPREE-Neuroimaging sub-study), hearing acuity measures (the ASPREE-Hearing sub-study) and retinal vascular calibre assessments (the ASPREE-AMD sub-study: Aspirin in Age-related Macular Degeneration; the ENVIS-ion study: neurovascular imaging). Each sub-study had specific inclusion and exclusion criteria in addition to those of the parent ASPREE trial.^20-23^

Ethics approvals were obtained from the Human Research Ethics Committee of Monash University for the parent ASPREE trial (registered on clinicaltrials.gov, number: NCT01028583) and for the four sub-studies registered on https://anzctr.org.au: The ASPREE-AMD (ACTRN12613000755730); The ENVIS-ion study (ACTRN12609000613202); The ASPREE-Hearing (ACTRN12614000496617); The ASPREE-Neuro (ACTRN12613001313729). A written consent form was obtained for each sub-study. This study conformed to the Declaration of Helsinki.

### Data collection

#### Routine assessment

At baseline and during in-person annual visits, anthropometric and laboratory measurements were collected, alongside data pertaining to medical morbidities, chronic conditions, lifestyle and socio-demographic factors, prescription medications and other relevant health variables. Details of the methods have been published previously.^24^

#### Neuroimaging

Individuals participating in the ASPREE-Neuro sub-study underwent an MRI scan using 3.0 Tesla Skyra scanner (Siemens, Erlangen, Germany) with a 32-channel head coil. Standardized sequences were used for brain morphometry, microstructure and function. T1-weighted MPRAGE (1 mm voxels) imaging was used to assess brain volume while Fluid-attenuated inversion recovery (FLAIR, 1.2 mm voxels) sequences were acquired to evaluate WMHs. WMH volumes were estimated using an automated lesion prediction algorithm, which was manually reviewed and edited for accuracy. Further segmentation of WMHs was carried out to distinguish between periventricular WMH (located within a distance of ≤8 mm from the lateral ventricles) and deep WMH (located more than 8 mm away from the ventricles).

#### Retinal vascular calibre

Data were extracted from the ASPREE-AMD and ENVIS-ion sub-studies. A detailed methodology of our retinal photography has been described previously.^21,23^ Retinal photography was undertaken using a non-mydriatic fundus camera (Canon Inc., Tokyo, Japan). The disc-centred image from one eye per participant was used for this analysis. The 6 largest arterioles and 6 largest venules located within a one and one-half diameter range from the optic disc margin were measured using a semi-automated computer system called ‘Interactive Vessel Analyzer’ (IVAN). IVAN included software for vascular calibre calculation, based on the Parr-Hubbard ‘big six’ formula.^25,26^ Averaged measures of retinal vessel diameters were presented as the central retinal arteriolar and venular equivalents (CRAE and CRVE).

#### Hearing acuity

Data were used from the ASPREE-HEARing sub-study.^20^ Briefly, air conduction measures were undertaken using portable audiometers (AD226; Interacoustics A/S) with ER3A fitted inserts and sound-attenuating earmuffs (Australian Standards AS/NZS 1270:2002). Sound detection thresholds were determined using pure tones at 0.5, 1, 2 and 4 kHz for each ear. An average of sound detection thresholds at 0.5, 1, 2 and 4 kHz in the better ear (termed 4FA) reflects the frequency range most relevant to speech audibility. A higher 4FA indicates poorer hearing acuity. All hearing thresholds were measured in decibels (dB).

The Listening in Spatialized Noise-Sentences Test was administered at each hearing visit to assess binaural speech perception in background noise.^27^ Results are expressed as the speech reception threshold (SRT), which is the difference in decibels between the target speech and background noise required for 50% word identification. A more negative SRT indicates better hearing performance.

### Outcome

The main outcome measure of this study was hearing acuity, defined as the mean sound detection thresholds for the low-frequency pure tone at 0.5 kHz, the high-frequency pure tone at 4 and 8 kHz, 4FA and SRT. Approximately 81% of the ASPREE-Hearing sub-study participants (250 of 308) completed their audiometry assessment at the year 3 follow-up study and they were included in the longitudinal analysis.

### Statistical analysis

Baseline characteristics were reported using descriptive statistics stratified by CRAE and CRVE quintiles (Q): Q1 (lowest 20%) represents the narrowest arteriole (CRAE) or venule (CRVE) and Q5 (highest 20%) represents the widest. Q2–Q4 were combined to create one group. Quintiles were used for retinal vessel calibres to align with established methodologies in large-scale vascular epidemiological studies such as the Multi-Ethnic Study of Atherosclerosis (MESA) and the Beaver Dam Offspring Study. Groups Q2–Q4 were combined to focus the comparison on the extremes of the distribution (Q1 versus Q5).

For white matter hyperintensity (WMH) volume, terciles were used due to the smaller sample size (*n* = 308) and the right-skewed distribution of WMH. WMH volumes as total, deep and periventricular were log-transformed to normalize the data prior to dividing into terciles, enabling meaningful comparison across low, medium and high burdens of WMH.

Cross-sectional analyses utilizing linear regression models were conducted to determine changes in hearing acuity with every 1 standard deviation (SD) difference in (i) baseline CRAE, (ii) baseline CRVE and (iii) baseline WMH volumes (total, deep and periventricular). Sex differences in baseline hearing measures were also explored. Additionally, linear regression models were used to determine the longitudinal association between a 1-SD difference in baseline CRAE, CRVE and WMH volumes on the change in hearing measures from baseline to year 3. Results were presented in β-coefficient and 95% confidence interval (CI). All models were adjusted for baseline confounding factors. Model 1 adjusted for age and sex alone and model 2 included the addition of key vascular risk factors that are important in the context of cerebral vessel disease (hypertension, diabetes, dyslipidaemia, smoking and eGFR). In the analyses with WMH volumes, the models were further adjusted for total brain volume (minus ventricles). A 2-sided *P* < 0.05 was used for determining statistical significance. Statistical analyses were performed using STATA software v17.0 (StataCorp LLC, College Station, TX, USA).

## Results

**Figure 1:**
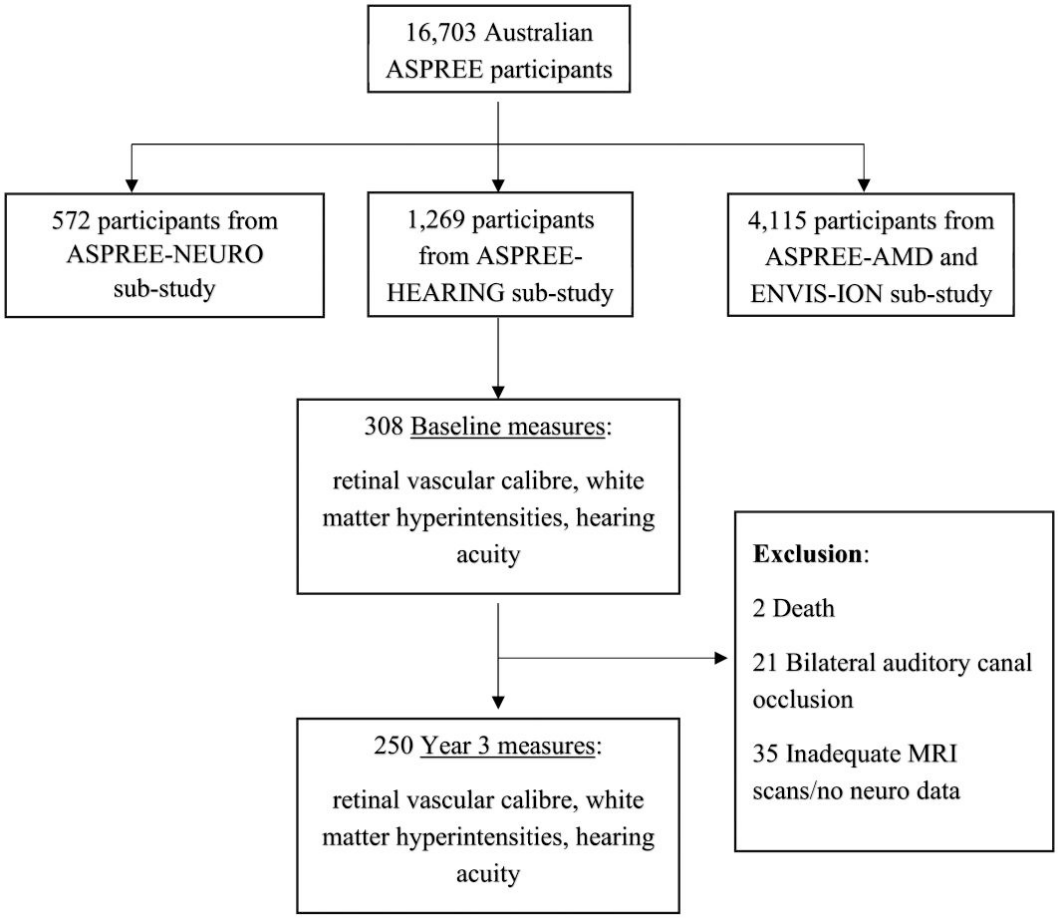
**Flow diagram of recruitment and sub-studies.** Of 16 703 Australian ASPREE participants, 308 had complete baseline data for retinal vascular calibre, white matter hyperintensity volume (from MRI), and hearing assessments; 250 completed follow-up at year 3. Exclusions (*n* = 58) included deaths (*n* = 2), bilateral auditory canal occlusion (*n* = 21) and inadequate MRI or missing neuroimaging data (*n* = 35). ASPREE, ASPirin in Reducing Events in the Elderly; MRI, magnetic resonance imaging; WMH, white matter hyperintensities.

Figure 1 outlines the process of selecting participants for the current study. Of 16 703 Australian ASPREE trial participants, 308 provided baseline measures for hearing, retinal vascular indices and WMHs. At the 3-year follow-up, data from 250 participants was available after excluding the deaths, cases of unmanaged auditory canal occlusion, and those lacking neuroimaging data.

### Study participants

Tables 1 and 2 present participant characteristics by quintiles of CRAE and CRVE calibres (distributions shown in Supplementary Fig. 3). The median age was 72.1 years (IQR, 71.1–74.7 years), with 155 men (50.3%). CRAE and CRVE calibres and WMH volumes are comparable to those reported from a similar community-dwelling older aged population, the MESA study, also free of cardiovascular disease at baseline.^28,29^

**Table 1:** Baseline characteristics of the study population according to quintiles of central retinal arteriolar equivalents (CRAE)

	CRAE		
	Q1 Mean (SD) = 125.0 (8.0)	Q2–4 Mean (SD) = 146.7 (7.2)	Q5 Mean (SD) = 167.5 (8.0)
	*N* = 63	*N* = 184	*N* = 61
Age (years), median (IQR)	71.8 (70.9–75.4)	72.3 (71.1–75.1)	72.3 (71.1–74.3)
Female, *n* (%)	31 (49.2%)	92 (50.0%)	30 (49.2%)
Smoker (% current)	3 (4.8%)	4 (2.2%)	1 (1.6%)
Alcohol (% current)	50 (79.4%)	158 (85.9%)	51 (83.6%)
BMI (kg/m^2^), mean (SD)	28.1 (4.3)	27.8 (4.2)	27.8 (4.7)
eGFRCKD (ml/min/1.73 m^2^), mean (SD)	72.8 (11.7)	77.6 (12.4)	78.1 (10.6)
Hypertension, *n* (%)	48 (76.2%)	135 (73.4%)	33 (54.1%)
Systolic blood pressure, mean (SD)	139.2 (17.9)	138.7 (15.3)	135.4 (16.1)
Diastolic blood pressure, mean (SD)	77.0 (8.7)	76.4 (9.7)	74.6 (10.1)
Diabetes, *n* (%)	8 (12.7%)	16 (8.7%)	6 (9.8%)
Dyslipidaemia, *n* (%)	35 (55.6%)	110 (59.8%)	40 (65.6%)
Total WMH (mm^3^), median (IQR)	4271 (2292–6630)	2941.5 (1769–5328)	3876 (1981–5627)
Deep WMH (mm^3^), median (IQR)	401 (173–1096)	252 (118.5–749.5)	324 (196–832)
Periventricular WMH (mm^3^), median (IQR)	3845 (2102–5887)	2553.5 (1553–4417)	3392 (1831–4830)

IQR, interquartile range; SD, standard deviation; BMI, body mass index; SBP, systolic blood pressure; DBP, diastolic blood pressure; CRAE, central retinal arteriolar equivalents (µm); WMH, white matter hyperintensity.

**Table 2:** Baseline characteristics of the study population according to quintiles of central retinal venular equivalents (CRVE)

	CRVE		
	Q1 Mean = 182.2 (11.1)	Q2–4 Mean (SD) = 213.4 (9.4)	Q5 Mean (SD) = 242.6 (12.6)
	*N* = 62	*N* = 185	*N* = 61
Age (years), median (IQR)	72.7 (71.4–76.7)	72.0 (71–74.3)	72.1 (71.1–74.6)
Female, *n* (%)	31 (50.0%)	92 (49.7%)	30 (49.2%)
Smoker (% current)	4 (6.5%)	3 (1.6%)	1 (1.6%)
Alcohol (% current)	51 (82.3%)	155 (83.8%)	53 (86.9%)
BMI (kg/m^2^), mean (SD)	27.4 (3.9)	27.6 (4.3)	28.9 (4.7)
eGFRCKD (ml/min/1.73 m^2^), mean (SD)	74.0 (13.8)	77.1 (11.5)	78.1 (11.7)
Hypertension, *n* (%)	46 (74.2%)	129 (69.7%)	41 (67.2%)
Systolic blood pressure, mean (SD)	138.8 (18.3)	138.0 (15.9)	138.2 (14.0)
Diastolic blood pressure, mean (SD)	75.6 (10.3)	76.2 (9.2)	76.7 (10.1)
Diabetes, *n* (%)	6 (9.7%)	19 (10.3%)	5 (8.2%)
Dyslipidaemia, *n* (%)	30 (48.4%)	111 (60%)	44 (72.1%)
Total WMH (mm^3^), median (IQR)	3331.5 (1981–5794)	3352 (2081–5818)	3224 (1633–4987)
Deep WMH (mm^3^), median (IQR)	259 (119–852)	294 (148–817)	278 (114–637)
Periventricular WMH (mm^3^), median (IQR)	3045 (1831–5003)	2885 (1799–4937)	2921 (1476–4588)

IQR, interquartile range; SD, standard deviation; BMI, body mass index; SBP, systolic blood pressure; DBP, diastolic blood pressure; CRVE, central retinal venular equivalents (µm); WMH, white matter hyperintensity.

Participants with the narrowest retinal arterioles (CRAE-Q1) were more likely to have a history of hypertension (76% versus 54% in Q5), less likely to have dyslipidaemia (56% versus 66% in Q5) and lower mean estimated glomerular filtration rate (eGFR) (73 versus 78 ml/min/1.73 m^2^) than those with the widest arterioles (Table 1). Those in the broader venular calibre group (Q5) were more likely to have dyslipidaemia (72% versus 48% in Q1), less likely have a history of hypertension (67% versus 74% in Q1) and slightly higher mean eGFR (78 versus 74 ml/min/1.73 m^2^) than those with the narrowest venules (Table 2). Total, deep and periventricular WMH volumes were comparable across CRAE and CRVE quintiles (Tables 1 and 2); corresponding values by WMH tercile are given in Supplementary Table 1 and their distributions in Supplementary Fig. 4.

### Cross-sectional differences: retinal vascular indices and hearing loss

Table 3 presents results from the linear regression models between a 1SD difference in CRAE, CRVE and WMHs on baseline hearing acuity. In the model adjusted for age and sex, a 1SD increase in CRAE revealed no change in the mean sound detection thresholds at 0.5 kHz (β-coefficient: 0.91, 95% CI = −0.36, 2.17), 4 kHz (β-coefficient: 0.05, 95% CI = −2.0, 2.1), 8 kHz (β-coefficient: 0.27, 95% CI = −2.2, 2.7) the 4FA (β-coefficient: 0.49, 95% CI = −0.90, 1.88), SRT (β-coefficient: −0.10, 95% CI = −0.52, 0.31). Further adjustments for additional vascular risk factors, taking aspirin and statins showed similar results.

**Table 3:** Cross-sectional analysis showing the β-coefficients and 95% confidence intervals between retinal vessel calibre, white matter hyperintensity volume and hearing acuity at baseline

	β-Coefficient (95% CI)	
	Model 1	Model 2
Retinal vessel calibres		
CRAE 1-SD difference		
0.5 kHz	0.91 (−0.36, 2.17)	0.88 (−0.44, 2.20)
4 kHz	0.05 (−2.0, 2.1)	−0.006 (−2.11, 2.09)
8 kHz	0.27 (−2.2, 2.7)	0.53 (−2.03, 3.09)
4FA	0.49 (−0.90, 1.88)	0.48 (−0.97, 1.94)
SRT	−0.10 (−0.52, 0.31)	−0.02 (−0.43, 0.39)
CRVE 1-SD difference		
0.5 kHz	0.71 (−0.55, 1.96)	0.69 (−0.62, 2.009)
4 kHz	0.89 (−1.1, 2.88)	0.87 (−1.20, 2.95)
8 kHz	−0.51 (−3.0, 1.9)	−1.18 (−2.71, 2.36)
4FA	0.64 (−0.74, 2.02)	0.68 (−0.76, 2.12)
SRT	0.04 (−0.37, 0.45)	0.10 (−0.31, 0.51)
White matter hyperintensities^a^		
Total WMH		
0.5 kHz	0.57 (−0.92, 2.05)	0.59 (−0.93, 2.12)
4 kHz	0.77 (−1.58, 3.12)	0.75 (−1.66, 3.16)
8 kHz	1.15 (−1.73, 4.03)	1.04 (−1.90, 3.97)
4FA	0.98 (−0.65, 2.60)	0.97 (−0.70, 2.65)
SRT	0.31 (−0.17, 0.79)	0.28 (−0.19, 0.75)
Deep WMH		
0.5 kHz	−0.34 (−1.21, 0.53)	−0.40 (−1.29, 0.50)
4 kHz	−0.18 (−1.55, 1.19)	−0.22 (−1.63, 1.19)
8 kHz	−0.37 (−2.05, 1.31)	−0.37 (−2.08, 1.34)
4FA	−0.23 (−1.18, 0.72)	−0.28 (−1.26, 0.71)
SRT	0.11 (−0.17, 0.39)	0.11 (−0.16, 0.39)
Periventricular WMH		
0.5 kHz	0.82 (−0.76, 2.40)	0.87 (−0.75, 2.49)
4 kHz	1.04 (−1.45, 3.54)	1.03 (−1.54, 3.59)
8 kHz	1.67 (−1.40, 4.73)	1.52 (−1.60, 4.64)
4FA	1.32 (−0.40, 3.05)	1.33 (−0.45, 3.11)
SRT	0.36 (−0.15, 0.86)	0.32 (−0.17, 0.82)

CRAE, Central Retinal Arteriolar Equivalents; CRVE, Central Retinal Venular Equivalents; WMH, white matter hyperintensity.

Beta-coefficients represent the mean change in hearing acuity relative to 1SD difference in CRAE (15.45 µm), 1SD change in CRVE (21.80 µm), 1 mm^3^ in the total WMH, Deep WMH and Periventricular WMH.

Model 1 adjusted for age, sex and education; Model 2 adjusted for age, sex, hypertension, dyslipidaemia, diabetes, smoking, eGFR, taking aspirin and statin.

^a^Models 1 and 2 (white matter hyperintensities analysis) additionally adjusted for total brain volume (minus ventricles).

Similarly, a 1SD increase in CRVE showed no significant changes across the hearing measures: 0.5 kHz (β-coefficient: 0.71, 95% CI = −0.55, 1.96), 4 kHz (β-coefficient: 0.89, 95% CI = −1.1, 2.88), 8 kHz (β-coefficient: −0.51, 95% CI = −3.0, 1.9), the 4FA (β-coefficient: 0.64, 95% CI = −0.74, 2.02), SRT (β-coefficient: −0.04, 95% CI = −0.37, 0.45). Further adjustments for additional vascular risk factor did not change the associations. Sex-stratified baseline hearing measures by CRAE and CRVE showed no statistically significant difference and can be found in Supplementary Table 2.

### Cross-sectional differences: white matter hyperintensity and hearing acuity

Table 3 also shows the association between 1 unit increase in log-transformed WMHs (as the total, deep and periventricular) and hearing thresholds (pure tone audiometry and speech reception). In both the basic (model 1) and fully adjusted models (model 2), a 1 unit difference in total WMHs showed no statistically significant association with mean sound detection thresholds. With the fully adjusted model total WMH results at 0.5 kHz (β-coefficient: 0.59, 95% CI = −0.93, 2.12), 4 kHz (β-coefficient: 0.75, 95% CI = −1.66, 3.16), 8 kHz (β-coefficient: 1.04, 95% CI = −1.90, 3.97), the 4FA (β-coefficient: 0.97, 95% CI = −0.70, 2.65), SRT (β-coefficient: 0.28, 95% CI = −0.19, 0.75) were unaffected by the additional adjustment. The associations remained non-statistically significant when dividing WMHs into deep and periventricular volumes.

Sex-stratified baseline hearing measures (different air conduction thresholds and SRT) by WMHs showed no statistically significant difference and can be found in Supplementary Table 3.

### Longitudinal changes: hearing acuity with WMHs and retinal vascular measures

As shown in Table 4, neither a 1SD increase in baseline CRAE nor CRVE was associated with changes in hearing acuity after 3 years. In the fully adjusted models, a 1SD increase in CRAE did not show a statistically significant change in hearing measures (0.5 kHz β-coefficient: 0.31, 95% CI = −1.24, 0.63), 4 kHz (β-coefficient: −0.08, 95% CI = −0.95, 0.78), 8 kHz (β-coefficient: 0.23, 95% CI = −1.27, 1.72), the 4FA (β-coefficient: −0.28, 95% CI = −0.94, 0.38), SRT (β-coefficient: 0.09, 95% CI = −0.20, 0.39). A 1SD increase in CRVE yielded similar results.

**Table 4:** Longitudinal analysis showing the β-coefficients and 95% confidence intervals (95% CIs) of the relationship between retinal vessel calibre, white matter hyperintensity volume and the 3 year change in hearing acuity from baseline

	β-Coefficient (95% CI)	
	Model 1	Model 2
Retinal vessel calibres		
CRAE 1-SD difference		
0.5 kHz	−0.48 (−1.37, 0.41)	0.31 (−1.24, 0.63)
4 kHz	−0.21 (−1.03, 0.61)	−0.08 (−0.95, 0.78)
8 kHz	−0.07 (−1.50, 1.35)	0.23 (−1.27, 1.72)
4FA	−0.42 (−1.05, 0.21)	−0.28 (−0.94, 0.38)
SRT	0.04 (−0.24, 0.32)	0.09 (−0.20, 0.39)
CRVE 1-SD difference		
0.5 kHz	0.03 (−0.85, 0.92)	0.27 (−0.64, 1.19)
4 kHz	−0.19 (−1.0, 0.63)	−0.07 (−0.92, 0.78)
8 kHz	−0.40 (−1.81, 1.00)	−0.37 (−1.84, 1.09)
4FA	−0.03 (−0.65, 0.60)	0.10 (−0.55, 0.74)
SRT	0.12 (−0.16, 0.39)	0.13 (−0.16, 0.42)
White matter hyperintensities^a^		
Total WMH		
0.5 kHz	0.26 (−0.76, 1.27)	0.07 (−0.96, 1.11)
4 kHz	0.57 (−0.36, 1.50)	0.48 (−0.48, 1.44)
8 kHz	0.85 (−0.76, 2.46)	0.62 (−1.03, 2.28)
4FA	0.41 (−0.31, 1.12)	0.27 (−0.46, 1.00)
SRT	0.04 (−0.28, 0.35)	0.01 (−0.31, 0.34)
Deep WMH		
0.5 kHz	0.36 (−0.22, 0.95)	0.30 (−0.29, 0.90)
4 kHz	0.27 (−0.24, 0.77)	0.25 (−0.27, 0.77)
8 kHz	0.51 (−0.42, 1.45)	0.41 (−0.55, 1.37)
4FA	0.28 (−0.13, 0.69)	0.22 (−0.19, 0.64)
SRT	0.03 (−0.16, 0.21)	0.002 (−0.19, 0.19)
Periventricular WMH		
0.5 kHz	0.10 (−1.0, 1.19)	−0.1` (−1.21, 1.00)
4 kHz	0.60 (−0.40, 1.60)	0.50 (−0.53, 1.54)
8 kHz	0.86 (−0.87, 2.58)	0.63 (−1.15, 2.40)
4FA	0.38 (−0.39, 1.14)	0.23 (−0.55, 1.01)
SRT	0.02 (−0.32, 0.36)	−0.003 (−0.35, 0.35)

CRAE, Central Retinal Arteriolar Equivalents; CRVE, Central Retinal Venular Equivalents; WMH, white matter hyperintensity.

Beta-coefficients represent the change in mean hearing acuity (from baseline to Year 3) relative to 1SD difference in CRAE (15.45 µm), 1SD change in CRVE (21.80 µm), 1 mm^3^ in the total WMH, Deep WMH and Periventricular WMH at baseline.

Model 1 adjusted for age, sex and education; Model 2 adjusted for age, sex, hypertension, dyslipidaemia, diabetes, smoking, eGFR, taking aspirin and statin.

^a^Models 1 and 2 (white matter hyperintensities analysis) additionally adjusted for total brain volume (minus ventricles).

Increases of 1 unit in baseline log-transformed total WMHs again showed no statistically significant change in hearing measures (in the fully adjusted model, 0.5 kHz β-coefficient: 0.07, 95% CI = −0.96, 1.11), 4 kHz (β-coefficient: 0.48, 95% CI = −0.48, 1.44), 8 kHz (β-coefficient: 0.62, 95% CI = −1.03, 2.28), the 4FA (β-coefficient: 0.27, 95% CI = −0.46, 1.00), SRT (β-coefficient: 0.01, 95% CI = −0.31, 0.34). The associations remained non-statistically significant when dividing WMHs into deep and periventricular volumes and adjusting for brain volumes.

The cross-sectional and longitudinal associations are also displayed visually in Supplementary Fig. 1 (cross-sectional) and Supplementary Fig. 2 (longitudinal).

## Discussion

This study examined whether ARHL is associated with concomitant changes in cerebral and retinal microvasculature in older adults aged 70 and above. The investigation was stimulated by results from previous studies reporting a link between retinal vascular calibre and hearing loss in younger cohorts.^13,15^ A strong association might allow the early stages of ARHL to be identified or predicted via non-invasive imaging of the eye or brain. However, the evidence to date has been inconsistent and ambiguous.

Our study found no significant associations between variations in the calibres of arterioles or venules and hearing acuity for either low- (0.5 kHz) or high-frequency (8 kHz) pure tones. Nor was an association found between retinal vascular indices and WMHs. Similarly, there was no relationship between WMH volumes—total, periventricular, or deep—and hearing acuity or SRT. The study was conducted in a primarily healthy older cohort.

Previous research on retinal vasculature and hearing acuity has focussed on younger populations where both retinal vascular characteristics and hearing loss are much less common.^13,15^ Amongst these studies the Child Health CheckPoint study in Australia, reported that microvascular differences including narrower arterioles and wider venules were linked to poorer hearing in mid-life, but the impairment was for low frequencies rather than the higher frequencies typical of ARHL.^13^ The Beaver Dam Offspring Study suggested that wider CRVE were associated with hearing impairment in mid-life adults, possibly as a result of both hearing impairment and retinal vascular differences being more common amongst those with cardiovascular risk factors.^15^ In the Atherosclerosis Risk in Communities (ARIC) study where the cohort had a mean age of 76.1 years, no association was found between CRAE and ARHL.^18^

The finding that higher white matter hyperintensity volumes are not related to hearing loss is significant because the presence of WMHs is closely related to cerebral small vessel disease and has been associated with other evidence of cerebral functional impairment.^30,31^ The results contrast with those of Eckert *et al*.,^17^ who identified a correlation between increased periventricular WMHs and reduced perception of low-frequency sounds in females with hypertension. Other studies have linked WMHs with difficulties in understanding speech, particularly in older adults with normal auditory thresholds.^16^

It is plausible that our null findings reflect the use of global WMH volume rather than region-specific lesions. Emerging evidence suggests that white matter integrity in the auditory cortex and associated pathways, particularly the inferior colliculus, medial geniculate body and Heschl’s gyrus, may be more closely linked to hearing function than global burden. For example, one study using diffusion tensor imaging has shown that reduced fractional anisotropy in the auditory radiations, an indication of disrupted white matter integrity, correlates with speech-in-noise perception, independent of peripheral hearing thresholds.^32^ Furthermore, age-related cortical thinning in the primary auditory cortex has been associated with degraded speech processing.^33^ Our analysis did not include regional WMH segmentation or measures of cortical thickness, which may have identified localized relationships between small vessel pathology and auditory decline. Future studies may employ voxel-based morphometry, tract-specific analyses, or region of interest approaches to explore whether WMHs in temporal lobe regions or disruptions in the central auditory pathway are more strongly associated with hearing impairment than global burden.

The study's strengths include its cross-sectional and longitudinal design and its focus on generally healthy, community-dwelling older adults, which minimizes the confounding influence of other major chronic illnesses. The use of sensitive and objective assessments for hearing and speech perception increases the validity of the findings. Limitations include the moderate sample size which may have been insufficient to detect more subtle associations between retinal vascular calibre, WMHs and hearing function. Similarly the relatively short follow-up period of 3 years might not capture long-term changes in these relationships given the progressive nature of ARHL. As discussed above, analysis of global WMHs, rather than specific changes in auditory cortex regions, may have overlooked potentially relevant localized effects.

## Conclusion

This study provides information concerning the relationship between auditory function, speech discrimination and cerebral microvascular characteristics in older adults. Contrary to previous reports, it found no significant relationship between retinal vascular calibres, WMHs and hearing acuity or speech perception in this population. These findings suggest that vascular differences contributing to hearing loss are localized and not directly reflected elsewhere in the vasculature of the eye or brain. It is also possible that declining auditory function in older adults may be influenced principally by other factors causing degenerative changes in the auditory pathway. Future research could benefit from focusing on specific auditory pathways and regional brain differences to better understand these relationships.

## Supplementary Material

fcag133_Supplementary_Data

## Data Availability

De-identified data are available upon request from the ASPREE Management System (https://ams.aspree.org/application/home.aspx). The analysis code supporting the findings of this study is available from the corresponding author upon reasonable request. D.P.Q.C, C.T. and J.J.M. had full access to all the data in the study and take full responsibility for the integrity of the data and accuracy of the data analysis.
