# Supplementary material for: White matter hyperintensities, retinal vascular calibre and changes in age-related hearing loss
Source: Brain Commun. 2026 Apr 15;8(2):fcag133. doi: 10.1093/braincomms/fcag133 (PMC13126660; doi:10.1093/braincomms/fcag133)
Supplement: fcag133_Supplementary_Data [file fcag133_supplementary_data.docx]

**SUPPLEMENTARY TABLE 1.** Characteristics of the study population according to total WMH volume.

|  | **Total WMH** | | |
| --- | --- | --- | --- |
|  | **T1**  Median (IQR) | **T2**  Median (IQR) | **T3**  Median (IQR) |
|  | N = 103 | N = 103 | N = 102 |
| Age (years), median (IQR) | 72.2 (71.0-73.9) | 71.7 (71.1-74.3) | 73.1 (71.1-77.4) |
| Female, n (%) | 51 (49.5%) | 51 (49.5%) | 51 (50%) |
| Smoker (% current) | 1 (1%) | 3 (2.9%) | 4 (3.9%) |
| Alcohol (% current) | 91 (88.4%) | 88 (85.4%) | 80 (78.4%) |
| BMI (kg/m^2^), mean (SD) | 28.1 (4.2) | 28.0 (4.4) | 27.4 (4.4) |
| eGFR~~-~~CKD (mL/min/1.73m^2^), mean (SD) | 76.8 (12.4) | 77.5 (11.1) | 75.7 (12.7) |
| Hypertension, n (%) | 70 (68.0%) | 70 (68.0%) | 76 (74.5%) |
| Systolic blood pressure, mean (SD) | 137.0 (15.1) | 137.5 (14.4) | 140.0 (18.3) |
| Diastolic blood pressure, mean (SD) | 74.6 (10.2) | 76.3 (9.8) | 77.6 (8.5) |
| Diabetes, n (%) | 11 (10.7%) | 11 (10.7%) | 8 (7.8%) |
| Dyslipidaemia, n (%) | 69 (67.0%) | 58 (56.3%) | 58 (56.9%) |
| CRAE (μm), mean (SD) | 146.8 (14.1) | 147.2 (15.2) | 145.1 (17.0) |
| CRVE (μm), mean (SD) | 214.4 (22.0) | 211.6 (22.1) | 212.8 (21.4) |

Abbreviations: IQR, interquartile range; BMI, body mass index; SBP, systolic blood pressure; DBP, diastolic blood pressure; WMH, white matter hyperintensity

**SUPPLEMENTARY TABLE 2**: Baseline hearing measures in quintiles of retinal vascular calibres, stratified by sex

|  | **CRAE- Males** | | | | **CRAE- Females** | | | |
| --- | --- | --- | --- | --- | --- | --- | --- | --- |
|  | **Q1**  **(n = 32)** | **Q2-4**  **(n = 92)** | **Q5**  **(n = 31)** | **P-value** | **Q1**  **(n = 31)** | **Q2-4**  **(n = 92)** | **Q5**  **(n = 30)** | **P-value** |
| **Vessel calibre (µm), mean (SD)** | 124.1 (9.0) | 145.5 (7.2) | 165.0 (6.1) |  | 126.0 (6.7) | 147.9 (7.1) | 170.1 (9.0) |  |
| **Air conduction** |  |  |  |  |  |  |  |  |
| 0.5kHz | 17.8 (9.7) | 19.1 (9.3) | 18.7 (10.2) | 0.71 | 16.1 (12.4) | 20.8 (13.2) | 18.0 (13.8) | 0.58 |
| 4kHz | 42.5 (19.7) | 45.1 (19.4) | 41.9 (17.1) | 0.91 | 28.9 (16.1) | 32.2 (17.8) | 31.5 (17.8) | 0.56 |
| 4FA | 26.2 (11.8) | 28.7 (11.9) | 27.1 (11.4) | 0.76 | 22.3 (12.7) | 25.5 (13.6) | 24.8 (14.7) | 0.48 |
| **SRT** | -9.9 (5.1) | -10.1 (3.5) | -10.5 (4.1) | 0.53 | -11.4 (4.3) | -11.2 (3.8) | -11.6 (2.4) | 0.90 |
|  | ***CRVE- Males*** | | | | ***CRVE- Females*** | | | |
|  | **Q1**  **(n = 32)** | **Q2-4**  **(n = 92)** | **Q5**  **(n = 31)** | **P-value** | **Q1**  **(n = 31)** | **Q2-4**  **(n = 92)** | **Q5**  **(n = 30)** | **P-value** |
| **Vessel calibre (µm), mean (SD)** | 184.3 (9.6) | 214.4 (9.0) | 243.7 (12.8) |  | 180.0 (12.2) | 212.5 (9.8) | 241.5 (12.6) |  |
| **Air conduction** |  |  |  |  |  |  |  |  |
| 0.5kHz | 20.2 (12.5) | 18.8 (8.5) | 17.5 (9.2) | 0.26 | 18.5 (13.2) | 19.6 (14.4) | 19 (9.3) | 0.90 |
| 4kHz | 44.4 (20.0) | 44.4 (18.7) | 42.3 (19.1) | 0.67 | 32.3 (17.4) | 30.9 (18.0) | 32.2 (15.8) | 0.98 |
| 4FA | 29.0 (13.5) | 27.9 (11.4) | 26.5 (11.1) | 0.41 | 24.8 (13.2) | 24.8 (14.7) | 24.3 (10.8) | 0.90 |
| **SRT** | -9.7 (4.2) | -10.2 (4.2) | -10.5 (2.9) | 0.41 | -11.2 (4.4) | -11.4 (3.8) | -11.2 (2.4) | 0.98 |

**Abbreviations:** 4FA = mean of pure tones at 0.5, 1, 2, and 4 kHz; SRT = speech reception threshold; CRVE = central retinal venular equivalent; CRAE = central arteriolar equivalent.

**SUPPLEMENTARY TABLE 3:** Baseline hearing measures in terciles of white matter hyperintensity, stratified by sex

|  | **Males (n = 155)** | | | | **Females (n = 155)** | | | |
| --- | --- | --- | --- | --- | --- | --- | --- | --- |
|  | **T1**  **(n = 52)** | **T2**  **(n = 52)** | **T3**  **(n = 51)** |  | **T1**  **(n = 51)** | **T2**  **(n = 51)** | **T3**  **(n = 51)** |  |
|  | **Total WMH** | | | | **Total WMH** | | | |
| **WMH (mm^3^), median (IQR)** | 1380 (899-1617) | 3223 (2580-3862) | 7182 (5549-10776) |  | 1670 (1184-2200) | 3436 (2989-4129) | 8268 (6337-11384) |  |
| **Air conduction** |  |  |  |  |  |  |  |  |
| 0.5kHz | 17.7 (8.2) | 19.5 (9.6) | 19.2 (10.8) |  | 17.3 (11.9) | 18.9 (13.9) | 21.7 (13.7) |  |
| 4kHz | 43.8 (18.4) | 43.3 (18.3) | 44.7 (20.5) |  | 27.6 (16.7) | 32.1 (18.6) | 34.5 (16.5) |  |
| 4FA | 26.6 (10.8) | 27.9 (11.7) | 29.1 (12.7) |  | 22.1 (13.1) | 25 (13.9) | 27.0 (13.6) |  |
| **SRT** | -10.5 (3.3) | -10.1 (4.6) | -9.8 (3.8) |  | -12.3 (3.0) | -11.3 (3.3) | -10.4 (4.4) |  |
|  | **Deep WMH** | | | | **Deep WMH** | | | |
| **WMH (mm^3^), median (IQR)** | 91 (35-141) | 277 (224-403) | 1082 (709-2594) |  | 83 (35-130) | 312 (251-426) | 14556 (1018-2468) |  |
| **Air conduction** |  |  |  |  |  |  |  |  |
| 0.5kHz | 17.1 (7.9) | 20.7 (10.7) | 19.0 (9.8) |  | 19.9 (13.0) | 18.5 (13.9) | 19.7 (13.1) |  |
| 4kHz | 42.3 (19.4) | 46.5 (18.4) | 42.8 (19.1) |  | 31.2 (18.1) | 30.6 (17.3) | 32.8 (16.8) |  |
| 4FA | 25.5 (10.4) | 30.3 (13.0) | 28 (11.7) |  | 24.9 (13.6) | 24.4 (13.9) | 25.0 (13.3) |  |
| **SRT** | -10.6 (4.4) | -10.0 (3.4) | -9.9 (4.2) |  | -11.8 (2.7) | -11.3 (3.7) | -10.8 (4.4) |  |
|  | **Periventricular WMH** | | | | **Periventricular WMH** | | | |
| **WMH (mm^3^), median (IQR)** | 1216 (769-1521) | 2657 (2356-3340) | 6019 (4711-9719) |  | 1507 (1146-2004) | 3030 (2698-3546) | 6463 (5224-9490) |  |
| **Air conduction** |  |  |  |  |  |  |  |  |
| 0.5kHz | 18.0 (8.5) | 19.1 (9.7) | 19.3 (10.5) |  | 17.2 (12.0) | 18.2 (13.3) | 22.5 (13.9) |  |
| 4kHz | 43.5 (18.1) | 42.6 (18.9) | 45.8 (20.2) |  | 27.5 (16.7) | 31.8 (19.4) | 35 (15.3) |  |
| 4FA | 26.4 (10.1) | 27.6 (12.2) | 29.6 (12.7) |  | 21.9 (13.2) | 24.6 (14.0) | 27.5 (13.3) |  |
| **SRT** | -10.6 (3.3) | -10.2 (4.6) | -9.7 (3.8) |  | -12.1 (3.1) | -11.6 (3.3) | -10.3 (4.4) |  |

**Abbreviations:** 4FA = mean of pure tones at 0.5, 1, 2, and 4 kHz; SRT = speech reception threshold; WMH = white matter hyperintensity

**SUPPLEMENTARY FIGURE 1.** Cross-sectional associations between retinal vessel calibre, white matter hyperintensity (WMH) volume and hearing acuity at baseline estimated using multivariable linear regression models. Points represent β-coefficients and horizontal bars indicate 95% confidence intervals. Two-sided t-tests used to assess the statistical significance of regression coefficients. None of the associations reached statistical significance (all p-values > 0.05)

Note: β -coefficients represent the mean change in hearing acuity relative to 1SD difference in CRAE (15.45µm), 1SD change in CRVE (21.80 µm), 1 mm^3^ in the total WMH, Deep WMH and Periventricular WMH.

Model 1 adjusted for age, sex and education; Model 2 adjusted for age, sex, hypertension, dyslipidaemia, diabetes, smoking, eGFR

*Models 1 and 2 (white matter hyperintensities analysis) additionally adjusted for total brain volume (minus ventricles).

Analyses were conducted at the participant level, with n = 308 participants included in each model. Panels show associations for (A) CRAE, (B) CRVE, (C) deep WMH, (D) periventricular WMH and (E) total WMH.

Abbreviations: CRAE, Central Retinal Arteriolar Equivalents; CRVE, Central Retinal Venular Equivalents; WMH, white matter hyperintensity


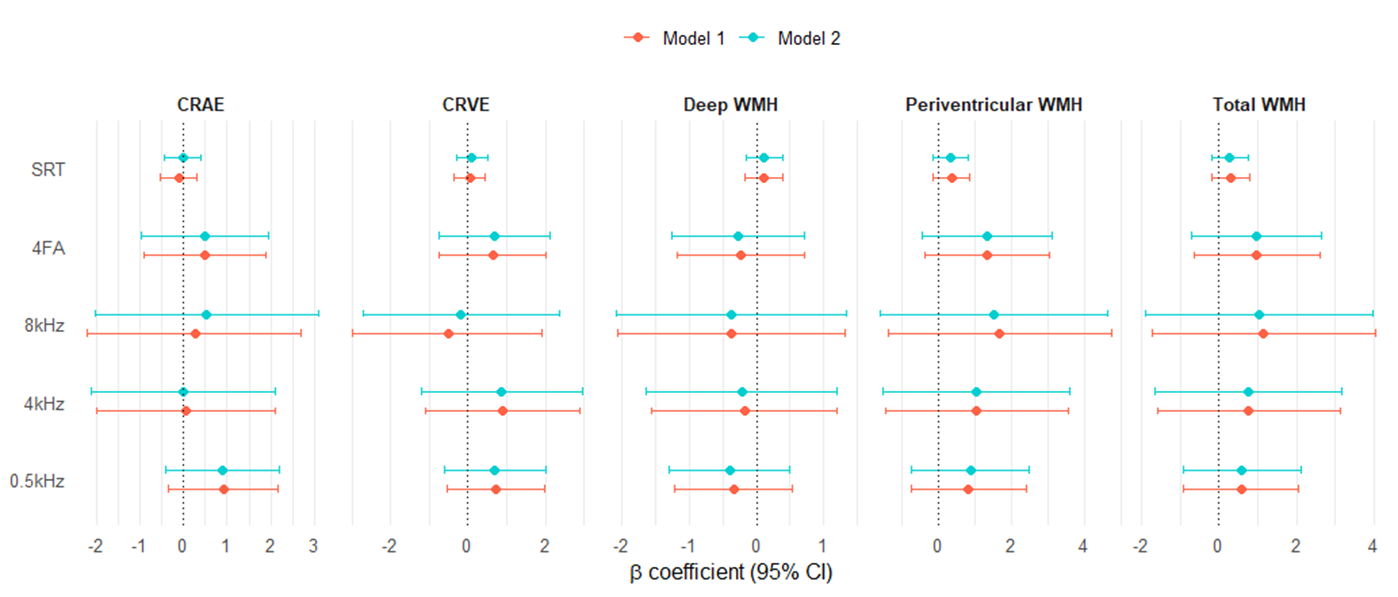


D

C

E

B

A

**SUPPLEMENTARY FIGURE 2.** Longitudinal analysis between retinal vessel calibre, white matter hyperintensity (WMH) volume and the 3-year change in hearing acuity from baseline estimated using multivariable linear regression models. Points represent β-coefficients and horizontal bars indicate 95% confidence intervals. Two-sided t-tests used to assess the statistical significance of regression coefficients. None of the associations reached statistical significance (all p-values > 0.05)

Note: β-coefficients represent the 3-year mean change in hearing acuity relative to 1SD difference in CRAE (15.45µm), 1SD change in CRVE (21.80 µm), 1 mm^3^ in the total WMH, Deep WMH and Periventricular WMH at baseline.

Model 1 adjusted for age, sex and education; Model 2 adjusted for age, sex, hypertension, dyslipidaemia, diabetes, smoking, eGFR

*Models 1 and 2 (white matter hyperintensities analysis) additionally adjusted for total brain volume (minus ventricles).

Analyses were conducted at the participant level, with n = 250 participants included in each model. Panels show associations for (A) CRAE, (B) CRVE, (C) deep WMH, (D) periventricular WMH and (E) total WMH.

Abbreviations: CRAE, Central Retinal Arteriolar Equivalents; CRVE, Central Retinal Venular Equivalents; WMH, white matter hyperintensity


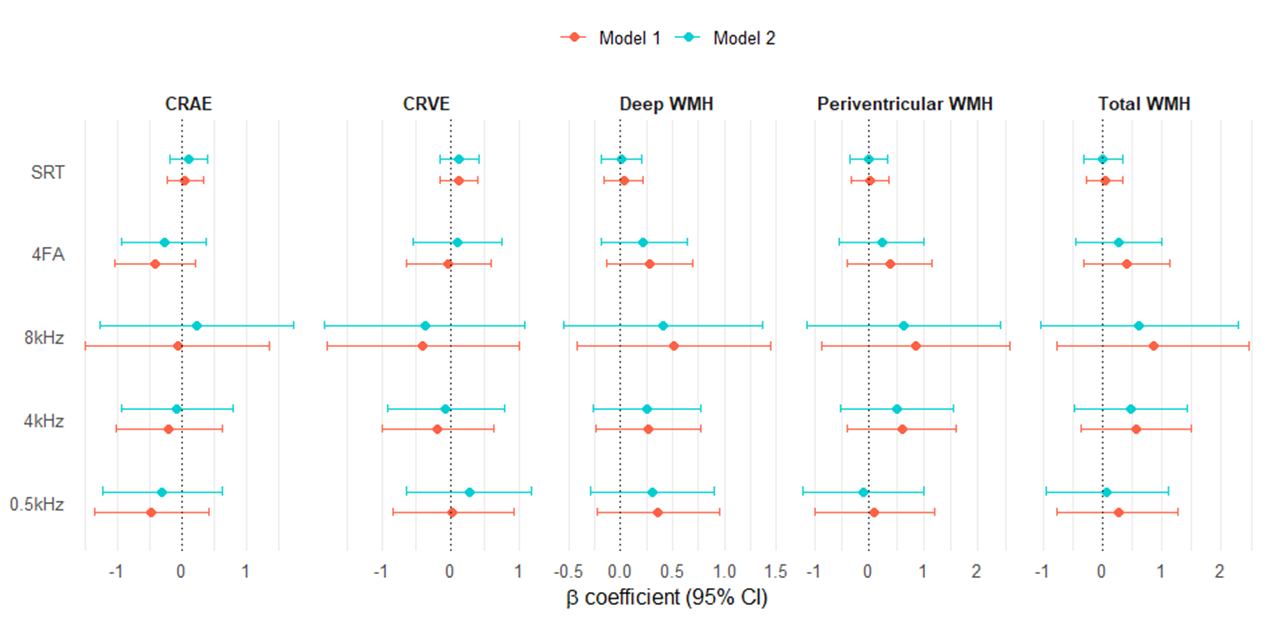


D

E

A

B

C

**SUPPLEMENTARY FIGURE 3:** Kernel density plot showing the kernel-density distributions of A) central retinal arteriolar equivalent (CRAE) and B) central retinal venular equivalent (CRVE) at baseline, with quintile cut-points indicated. Analyses were conducted at the participant level, with n = 308 participants included in each model. Abbreviations: CRAE, Central Retinal Arteriolar Equivalent; CRVE, Central Retinal Venular Equivalent.


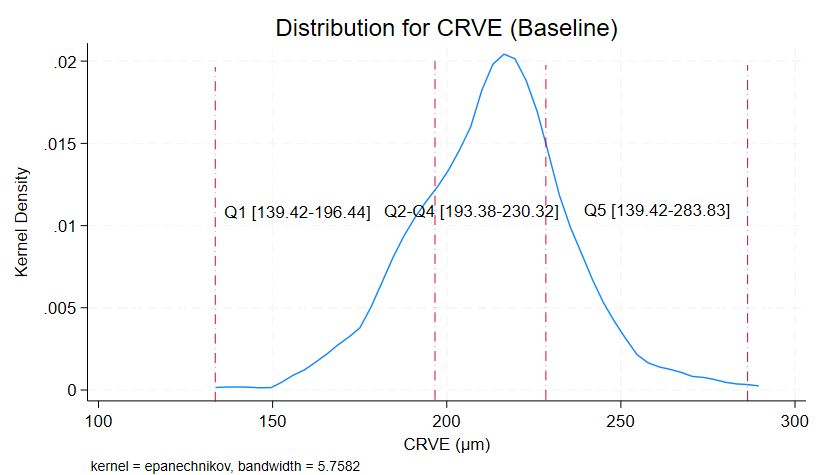

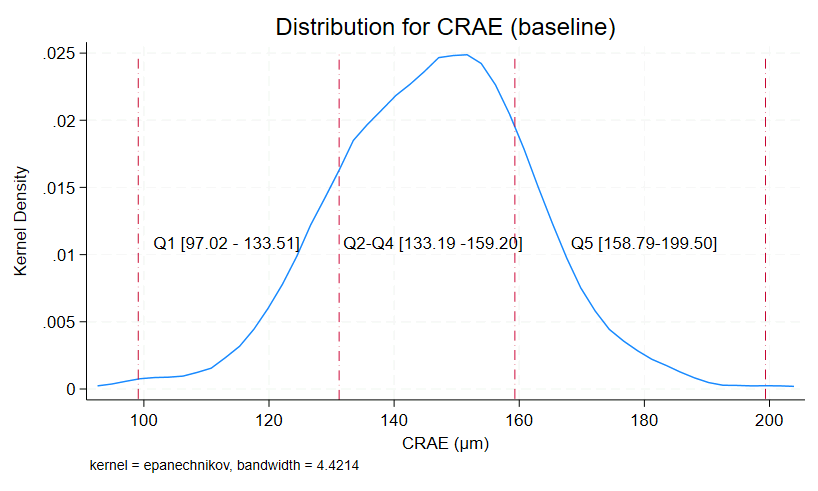


B

**SUPPLEMENTARY FIGURE 4:** Kernel density plot showing the kernel- density distributions of white matter hyperintensity volumes (A) Total, B) deep & C) peri-ventricular) at baseline. Analyses were conducted at the participant level, with n = 308 participants included in each model. Abbreviations: WMH, white matter hyperintensity


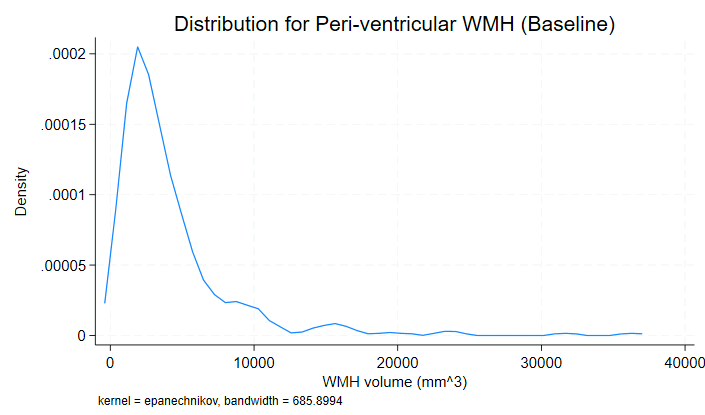

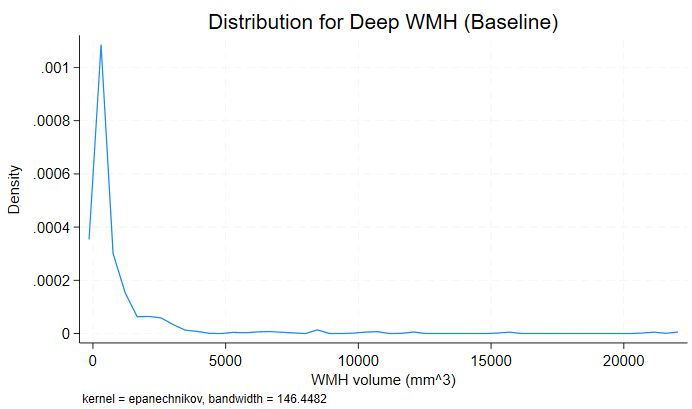

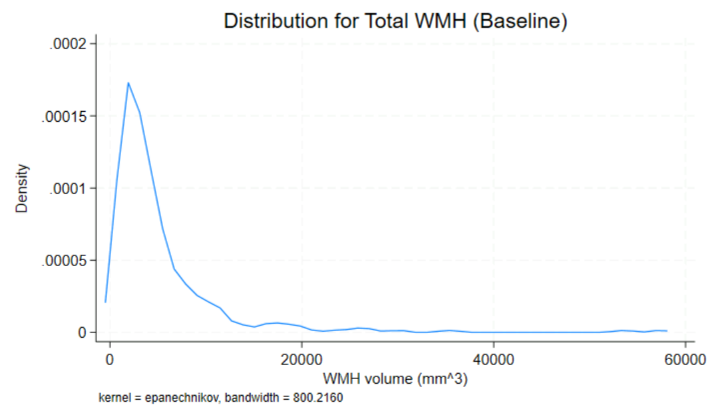


C

B

A
